# Obesity and adipose tissue impact on T-cell response and cancer immune checkpoint blockade therapy

**DOI:** 10.1093/immadv/ltac015

**Published:** 2022-06-24

**Authors:** Gabriel Pasquarelli-do-Nascimento, Sabrina Azevedo Machado, Juliana Maria Andrade de Carvalho, Kelly Grace Magalhães

**Affiliations:** Laboratory of Immunology and Inflammation, Department of Cell Biology, University of Brasilia, DF, Brazil; Laboratory of Immunology and Inflammation, Department of Cell Biology, University of Brasilia, DF, Brazil; Laboratory of Immunology and Inflammation, Department of Cell Biology, University of Brasilia, DF, Brazil; Laboratory of Immunology and Inflammation, Department of Cell Biology, University of Brasilia, DF, Brazil

**Keywords:** immunotherapy, obesity, cancer, adipose tissue

## Abstract

Many different types of cancer are now well known to have increased occurrence or severity in individuals with obesity. The influence of obesity on cancer and the immune cells in the tumor microenvironment has been thought to be a pleiotropic effect. As key endocrine and immune organs, the highly plastic adipose tissues play crucial roles in obesity pathophysiology, as they show alterations according to environmental cues. Adipose tissues of lean subjects present mostly anti-inflammatory cells that are crucial in tissue remodeling, favoring uncoupling protein 1 expression and non-shivering thermogenesis. Oppositely, obese adipose tissues display massive proinflammatory immune cell infiltration, dying adipocytes, and enhanced crown-like structure formation. In this review, we discuss how obesity can lead to derangements and dysfunctions in antitumor CD8+ T lymphocytes dysfunction. Moreover, we explain how obesity can affect the efficiency of cancer immunotherapy, depicting the mechanisms involved in this process. Cancer immunotherapy management includes monoclonal antibodies targeting the immune checkpoint blockade. Exhausted CD8+ T lymphocytes show elevated programmed cell death-1 (PD-1) expression and highly glycolytic tumors tend to show a good response to anti-PD-1/PD-L1 immunotherapy. Although obesity is a risk factor for the development of several neoplasms and is linked with increased tumor growth and aggressiveness, obesity is also related to improved response to cancer immunotherapy, a phenomenon called the obesity paradox. However, patients affected by obesity present higher incidences of adverse events related to this therapy. These limitations highlight the necessity of a deeper investigation of factors that influence the obesity paradox to improve the application of these therapies.

## Obesity and adipose tissue

Obesity is currently considered a devastating pathology as it increases the individual risk for a plethora of diseases, such as heart disorders, dyslipidemia, chronic kidney disease, hypertension, type 2 diabetes (T2D), stroke, and cancer, and display intimate association with insulin resistance (IR) and chronic low-grade inflammation progressive worsening [1]. World Health Organization (WHO) defines overweight and obesity in terms of a body mass index (BMI), higher or equal to 25 kg/m^2^ and higher or equal to 30 kg/m^2^, respectively, and a significant increase in obesity’s prevalence has been of great concern worldwide [2]. The latest WHO’s global health report addressed that the prevalence of obesity has increased 50% from 2000 to 2016. Specifically, more than 1.9 billion adults were overweight in 2016 and of these 650 million adults were obese globally [3]. Previously, obesity was described as being solely an energy imbalance over time, due to excessive energy storage when compared with energy expenditure, hence accumulating adipose tissue (AT) [4]. Nonetheless, obesity is now seen as a result of AT dysfunctional expansion and as a multifactorial disease, since AT is a highly active endocrine organ, playing a central role in the orchestration of metabolic and immune homeostasis [5]. The augmented adiposity is related to alterations in the tissue physiological profile. Obesity-related dysfunctional expansion of fat depots results in hyperinsulinemia, dyslipidemia with an excessive release of free fatty acids (FFA), and adipocyte hypoxia, inadequate oxygen supply, thereby promoting cell death, and the accumulation of proinflammatory mediators [6].

Pre-adipocytes, fibroblasts, endothelial cells, immune cells, and mature adipocytes are the main cell types that constitute AT [7]. Four distinct types of adipocytes have already been described: white, beige, brown, and pink [8]. These cells differ in morphological and physiological characteristics. Brown adipose tissue (BAT) and white adipose tissue (WAT) are the most well-described types of AT in the literature because they are constitutively present in the organism. White adipocytes, predominant in WAT, are marked by a unilocular lipid droplet, smaller and less abundant mitochondria, and are classically associated with energy intake signaling and storage [9]. In contrast, BAT is mainly composed of brown adipocytes, characterized by their multilocular lipid droplets and high amounts of mitochondria, which express elevated levels of uncoupling protein 1 (UCP1). Accordingly, BAT is implicated in heat production (non-shivering thermogenesis) and weight loss, and showing modifications throughout the individual’s life [10].

BAT is maximally recruited in birth, when it shifts drastically from a quiescent form into its most activated state in terms of heat production to provide thermoregulation, being located in the interscapular region in newborns and small mammals [11]. During childhood, BAT is reduced, since the individuals have already been exposed to cold and start a skeletal muscle shivering heat production. Evidence indicate that there may be a transient increase in BAT’s volume near puberty [12], suggesting that BAT is implicated in other functions besides the replacement of an absent shivering thermogenic process. In adulthood, there is reduced detection of basal BAT activity and cold-stimulation of this tissue may significantly decline since middle age, along with a reduced expression of UCP1 [13]. Noteworthy, BAT also tend to morphologically change over time toward a more prominent lipid storage profile, emphasizing the changes in BAT’s composition and function throughout mammals’ lives [14].

ATs are extremely plastic and, thus, have the capability of expanding and remodeling. The expansion of ATs can be a result of hypertrophy, hyperplasia, and adipocyte differentiation and is sensitive to external factors, such as nutrition and exercise routines. AT types can undergo transdifferentiation. BAT can convert into an intermediate beige phenotype, through a process called whitening [15]. The inverse process also occurs, the conversion of WAT into brown-like AT is the result of a process called browning. Browning occurs in response to cold exposure, augmented physical exercise, the action of pharmacological molecules, and changes in diet itself [16]. This process is regulated by the influence of sympathetic nerves on β-adrenergic receptors, triggering an intracellular signaling cascade. Alterations in cAMP levels can be detected by protein kinase A (PKA), which leads to the activation of mitogen-activated protein kinases, culminating in catabolic reactions that convert triglycerides (TGs) into fatty acids (FAs) and also in the activation and upregulation of UCP1, which allows the uncoupled production of ATP and heat dissipation [17]. Currently, browning and BAT activation have been studied as promising therapeutic strategies against obesity [18].

## Immune regulation in adipose tissue

Current research describes ATs as heterogeneous organs that regulate immunity, metabolism, and inflammation [19]. As studies show, both AT residing and infiltrating immune cells influence the inflammatory and metabolic status systemically and at tissue level. Diving into the mechanisms by which immune cell signaling modulates ATs’ function must occur to understand their influence on the onset, progression and treatment of many diseases [20]. Although efforts have been made in recent years, there is still scarce information regarding the characteristics of BAT immune cells. Macrophages and monocytes, eosinophils, B lymphocytes, and T regulatory (Treg) cells are present in BAT [21]. These immune cells are also key components in WAT architecture and, with unconventional lymphocyte subtypes innate lymphoid cells (ILCs), invariant natural killer T (iNKT) cells, and γδ T cells, influence WAT physiology [22–24].

Eosinophils residing in ATs are known to be sustained by IL-5 and CCL11 (eotaxin 1) tissue levels [25]. In addition to their crucial roles in allergic diseases and in the response against helminth infections, eosinophils play important roles in AT homeostasis maintenance, supporting type 2 (Th2)-associated immune profiles [26]. As described by Hasty and colleagues, macrophages represent only 30% of BAT’s leukocyte population [27]. B lymphocytes make up 20–30% of BAT immune cells and are regulated through the norepinephrine-beta2-adrenergic receptor (β2AR) interaction [27, 28]. In addition to its anti-inflammatory properties, Treg was described to display a BAT-specific phenotype that favors proper tissue function during cold exposure, as absence copes with impaired thermogenesis and diminished oxygen consumption [29].

Also described in lean WAT, the Treg cell is one of the main actors in controlling tissue inflammation and modulating its plastic phenotype [30]. In subcutaneous WAT (scWAT), activated Treg increases anti-inflammatory markers and augments the tissue thermogenic capacity through increasing UCP1 expression [31]. Lean visceral WAT (vWAT) is associated with a Treg profile that is preferentially adapted to fatty acid (FA) metabolism, the visceral adipose tissue Treg [32]. B lymphocytes also present a regulatory cell subset (Breg), which, in addition to secreting antibodies, restrain pathological tissue inflammatory responses through the secretion of interleukin-10 (IL-10) and transforming growth factor-beta (TGF-β) [33]. Treg and Breg immunomodulatory activity favor AT accumulation of alternatively activated macrophages (M2), which, through type 2 cytokine secretion, aid in conserving tissue immune and metabolic homeostasis. In addition, M2 participates in AT vasculogenesis and remodeling processes [34]. Also, a key factor in AT homeostasis, CD4+ T lymphocytes (T Helper 2-Th2) are known to orchestrate anti-inflammatory immune profile in ATs, inhibiting the occurrence of an immunologically harsh environment, and favoring adequate wound healing [35].

The innate-like T cells, iNKT cells, are enriched in ATs of humans and mice and account for 15–20% of total T cells [36]. Regulatory iNKT influences other anti-inflammatory immune cells homeostasis and avoid tissue inflammation through secretion of Th2-associated cytokines, including interleukin-5 (IL-5) and IL-13 [23]. Also linked to elevated anti-inflammatory cytokine levels, active innate lymphoid cell 2 (ILC2) stimulate beige fat biogenesis [37]. Another unconventional T lymphocyte, γδ T cells, are enriched in ATs and also present immunomodulatory roles and are crucial for promoting sympathetic innervation, as ablation of these cells impairs this process [38].

## Obesity-associated adipose tissue inflammation and system physiology derangements

**Figure 1.**
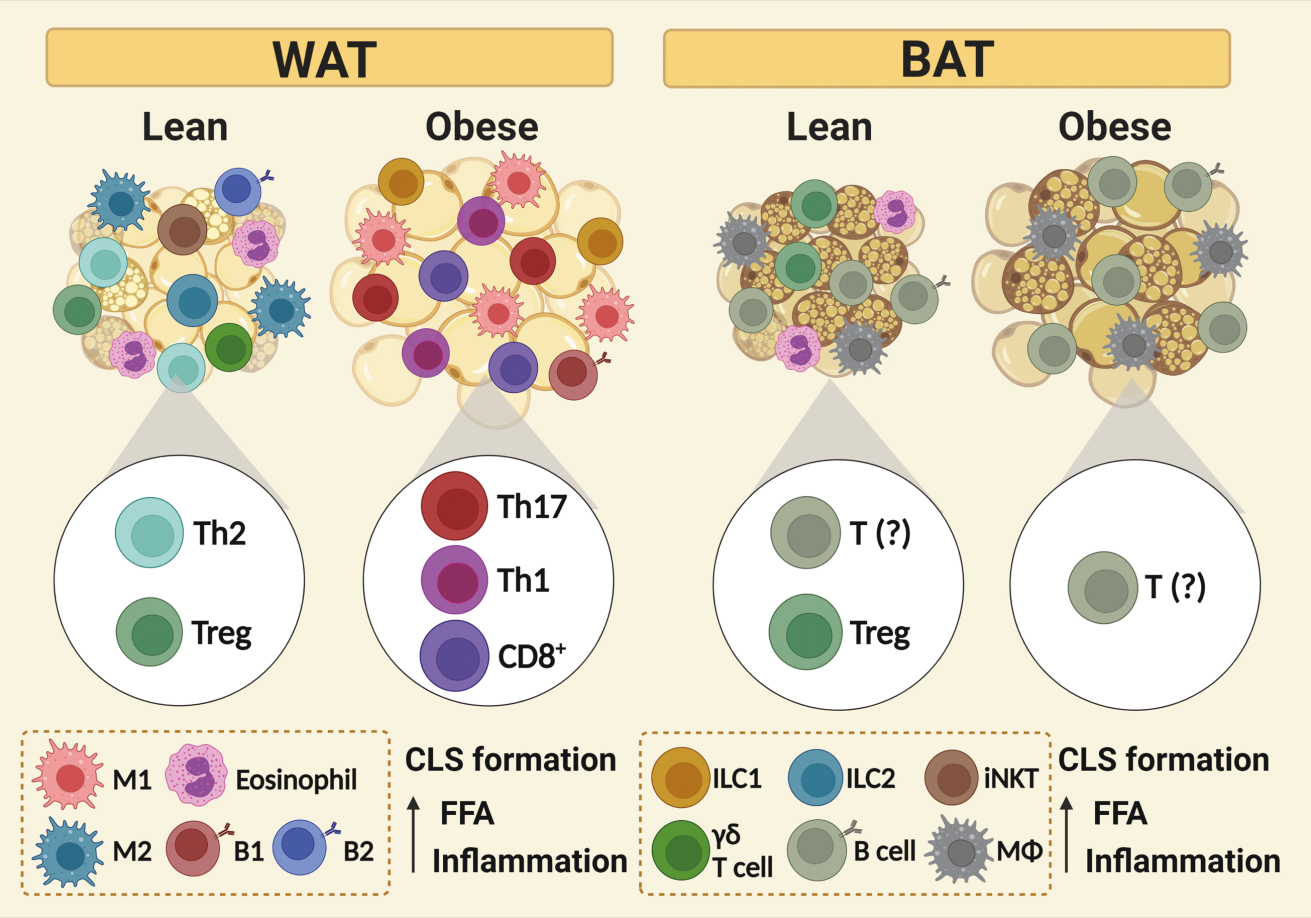
Immune cells from BAT and WAT of lean and obese phenotypes. While WAT of lean individuals tends to present mostly anti-inflammatory polarized immune cells (including Th2, Treg, and ILC2) WAT associated with obese phenotypes shows tissue inflammation due to an imbalance favoring proinflammatory immune cells, especially T lymphocytes (CD8+, Th1, Th17), B cells (B1) and macrophages (M1), which together form crown-like structures (CLS) and favors FFA release. Although BAT is less explored in the literature in this context and therefore is not as well characterized as WAT, T regulatory cells (Treg) and other anti-inflammatory immune cells have been identified in lean BAT. Obese BAT also presents inflammatory activity coped with massive infiltration of macrophages (MΦ), B cells, and T lymphocytes, although their polarization profile needs yet to be defined. The image emphasizes the most abundant cell types but is important to highlight that all AT types and phenotypes exhibit all cited immune cells.

During homeostasis, ATs show an increased number and activation of type 2 cytokine-secreting immune cells [35]. In contrast, the ATs associated with the obese phenotype present tissue inflammation due to proinflammatory profiles displayed by residing and infiltrating immune cells [39] (Fig. 1). BAT’s function is impaired during obesity, as cold-induced thermogenesis, insulin sensitivity, and glucose consumption are disrupted in the BAT of subjects affected by obesity [40]. In an elegant study, Alcala and colleagues informed that diet-induced obese mice showed BAT dysfunction due to the infiltration of immune cells (including lymphocytes and macrophages), cytokine release, and oxidative stress [41]. Another important study confirmed that B cells from BAT of individuals with obesity are increased in number [27].

B lymphocytes are also enriched in obese WAT, are associated with higher levels of proinflammatory mediators and decreased amounts of anti-inflammatory cytokines, and with the secretion of pathogenic IgG [42]. CD8- and CD4-expressing cells also play key roles in the induction of AT inflammation in the context of obesity. Effector CD8+ T lymphocytes are known for mediating antiviral and antitumor immunity by inducing apoptosis in other cells and systemically secreting the proinflammatory cytokine interferon (IFN)-γ [43]. Antibody depletion of CD8+ T cells from obese models decreases vWAT inflammation and increases insulin sensitivity, while adoptive transfer of these cells to CD8-deficient mice copes with proinflammatory mediators’ secretions, IR, and glucose intolerance [44]. CD4+ T cells (Th1) drive type 1 cytokine release (as IFN-γ) in ATs of individuals with obesity, amplifying tissue chronic inflammation [45]. Shirakawa and others linked a unique subpopulation of CD153+PD-1+CD44hiCD4+ T lymphocytes with WAT inflammation and systemic insulin resistance under influence of an obesogenic diet and affirmed that these cells accumulate in obese WATs [46]. The saturated FA palmitate, common in the high-fat diet, dose-dependently activates T cells, favoring cytokine secretion, and reactive oxygen species generation [47]. After re-stimulation, T cells from ATs present higher inflammatory cytokine secretion and cells derived from subjects with obesity display decreased T-cell receptor (TCR) diversity [48, 49]. In the context of the obese phenotype, CD8+ T cells and Th1 cells favor macrophage M1 differentiation and proliferation. M1 macrophages make up over 50% of WATs’ immune cells in individuals with obesity. They increase in number according to white adipocyte size and total adiposity and display a unique metabolically activated phenotype associated with inflammation and metabolic markers [50]. During obesity, ILC1 also accumulates in WAT depots, produces IFN-γ, and contributes to the occurrence of tissue and systemic inflammatory processes and impaired insulin signaling, as cell adoptive transfer led to exacerbated metabolic disorder and proinflammatory macrophage polarization [51].

The inflammatory processes that take place in the context of obesity can also be explained by the decreased number of anti-inflammatory cells. The protective roles against obesity presented by Treg cells, ILC2, iNKT, Breg, and δγ-T cells highlight the inflammatory nature of obesity [24]. The fact that inflammatory molecules inhibit noradrenergic signaling links obesity to decreased thermogenic capacity [52]. Obesity-associated BAT disruption copes with tissue ‘whitening’ due to elevated circulating IFN-γ levels [53]. During BAT whitening, the tissue shows increased inflammatory and lipogenic markers, displays diminished UCP1 expression and thermogenesis, and presents crown-like structure (CLS) formation [53].

CLS, considered histological hallmarks of AT inflammation, are also found in inflamed WAT and consists of M1 macrophages and proinflammatory B (B1) and T lymphocytes (Th1 and Th17) surrounding stressed and dying adipocytes [54]. During obesity-induced stress, white and brown adipocytes secrete unbalanced levels of adipokines (augmented proinflammatory and diminished anti-inflammatory mediators), including low levels of adiponectin and of BAT’s adipokines as Fibroblast growth factor 21 (FGF21) and C-terminal fragment of SLIT2 protein (SLIT2-C), also increased amounts of BAT mediators such as Chemerin and Endothelin-1 (ET-1), and WAT secreted leptin [55]. Peaking leptin levels and resistance correlate with AT’s mass and BMI, hence, stimulating systemic increased levels of inflammation mediators, such as a proinflammatory adipokine [56].

The obese state is also connected with brown and white adipocyte subcellular alterations, including mitochondrial dysfunction and endoplasmic reticulum (ER) stress, due to the harsh environment characteristic of obesity [57]. Nutrient overload favors reactive oxygen species (ROS) production, which leads to mitochondrial dysfunction, further exacerbating intracellular oxidative stress [58]. Excessive food intake induces ER stress as well, a process that also leads to ROS biogenesis [59]. The intracellular detrimental effects of oxidative stress are connected with damage-associated molecular patterns release, AT dysfunction, and inflammation [57]. Noteworthy, many food components often associated with obesogenic diets, such as saturated FAs and processed sugars, induce adipocyte ER stress and inflammation [60]. These intracellular disturbances favor cell death pathways leading to FA massive release and proinflammatory immune cell activation [61].

Another trigger for ER and systemic inflammation in ATs is the endotoxin lipopolysaccharide, a cell wall component of specific intestine-dwelling bacteria, that shows peaking amounts in the bloodstream of individuals affected by obesity [62]. Many studies inform that these augmented endotoxin levels are due to intestinal dysbiosis characterized by decreased microbiota diversity and outgrowth of pathobionts which results in increased intestinal permeability [63]. Endotoxemia exacerbates the inflammatory status of subjects with obesity, further impairing insulin signaling and systemic metabolism [64].

Nutrients obtained from diet not only impact intestinal microbiome composition and diversity but also may pose systemic immunomodulatory regulation [65]. In conjunction with cytokines and transcription factors expression, glycemic levels influence T-cell fate [66]. T cells present sensing systems that detect nutrient availability alterations, which influence cell function and differentiation status [67]. Hyperglycemia and TCR activation lead T cell to up-regulate glucose uptake through glucose receptor 1 (GLUT1) and reprogram its metabolism toward glycolysis and glutaminolysis [68]. However, current research points out that this metabolic reprogramming has effects on T lymphocyte differentiation. Increased glycolysis favors CD4+ T lymphocytes to differentiate to either Th1 or Th17 immune profiles, depending on the use and activation of hypoxia-inducible factor 1α (HIF1α) [69]. CD8-expressing T cells are pictured according to a spectrum of glycolytic capacity, being CD8+ effector T (Teff) lymphocytes the cell that preferentially metabolizes glucose [70]. The adipokine leptin was shown to further enhance GLUT1 presence in the T-cell membrane, favoring T lymphocyte activation in hyperglycemic scenarios [71]. Studies inform that hyperglycemia drives activation and cytokine secretion by Th1, Th17, and CD8+ Teff [72]. These points help to explain why elevated glucose levels and hyperleptinemia are linked with higher levels of proinflammatory mediators in individuals affected by obesity.

In contrast, the anti-inflammatory Treg and Breg cells show a preferential requirement for FA oxidation and show higher resistance to lipotoxicity [73]. However, hyperinsulinemic environments impair IL-10 secretion by these cells and diminish their capacity of inhibiting TNF-α production by macrophages [74]. Therefore, hyperglycemic microenvironments rich in insulin, TCR ligands, and leptin, such as the ones associated with obesity and T2D, tend to result in inflammatory and metabolic diseases that impact the individual risk of developing other chronic co-morbidities.

The inflamed phenotype associated with obesity is also related to degrading effects on immunity. Diet-induced obesity and inflammation result in premature thymic involution, which leads to diminished production of T lymphocytes in the thymus, displaying decreased TCR diversity [75]. Obesity also favors adipocyte accumulation in the bone marrow, a process that disrupts hematopoiesis [76]. In addition, obesity is connected with disturbed secondary lymphoid organ function and less effective dendritic cell-mediated T-cell stimulation [77]. All these physiological consequences of obesity were reported to be linked with T lymphocyte senescence [78]. Human senescent (CD28−CD57+) CD8+ T lymphocytes occur in the obese and diabetic states and show ROS intracellular accumulation and cytokine secretion. Liver senescent CD8+ T lymphocytes are associated with mouse and human increased insulin resistance, inflammation, and adoptive transfer of senescent CD8+ T lymphocytes was shown to deteriorate systemic glucose homeostasis [78]. These dysfunctional T cells are related to impaired tumor surveillance, as indicated by Petrelli and other meta-analysis studies [79].

## Obesity and cancer

As depicted in the previous section, the obese state copes with derangements in inflammation, metabolism, and immunity, including the immune responses against tumors. A special report conducted by the International Agency of Research on Cancer (IARC) based on over 1000 epidemiologic studies demonstrated that there is an increased risk of developing at least 13 types of cancer in individuals with excess body WAT, including breast, liver, pancreas, and ovaries malignancies [80]. According to the Centers for Disease Control and Prevention (CDC), overweight and obesity-related cancer represented about 40% of cancers diagnosed in the United States, which corresponds to 630,000 people diagnosed only in 2014. In counterpoint to overall cancer incidence, which declined since the 1990s, obesity-related cancers increased. That data does not include colorectal cancer, which, alone, increased by 7% between 2005 and 2014 [81]. Globally, a statistical data point out that 481,000 new cancer diagnoses were related to obesity, designating excessive body adiposity as a well-established risk factor for cancer development [82].

Considering the categorization of obesity as a pandemic and, in parallel, the increased incidence of obesity-related cancers, studies on the influence of obesity on the outcomes of available antineoplastic therapies are increasing [83]. The world has experienced a decrease in cancer mortality rate due to early diagnosis and cancer treatments available and under development. Data from 2019 indicate that approximately 17 million Americans diagnosed with cancer were still alive by January 1st of that year. Fifty-six percent of these patients were diagnosed and properly treated within the previous 10 years. Several therapies are currently used, such as surgeries, radiotherapy, hormonal therapies, chemotherapies, and immunotherapies. These approaches can be sufficient alone or can be administered in combination depending on the clinical picture exhibited by the patient [84]. In the same way that the excess adiposity promotes differential prognoses depending on the tumor type and stage, the abnormal accumulation of body fat can promote differential results depending on the chosen therapeutic approach. Currently, it is known that obesity can affect treatments differently, however, there is no proper guidance for the management of oncologic patients with increased BMI under distinct therapies [85].

Patients with increased BMI have demonstrated a negative impact on the outcomes of conventional therapies such as cancer surgery, radiotherapy, and chemotherapy. Cancer surgery is a therapeutic approach that can act as a preventive measure or as a treatment. It is well known that obesity is a factor that predisposes the individual to wound complications, considering that adipose expansion is associated with less tissue vascularization, alteration of immune population, greater pressure under the tissue, and consequently a deficient wound healing process [86]. Furthermore, the impact of high adiposity on radiotherapy has indicated that an elevated BMI was positively related to lower efficiency of radiotherapy and clinical recurrence of prostate cancer, and overall mortality [87]. In breast cancer patients, a high BMI was associated with post-radiotherapy effects, such as dermatitis [88].

Chemotherapy is currently a standard cancer treatment, but the scenario is not so favorable for patients with obesity, since these individuals have poor outcomes for breast, prostate, endometrial, and colorectal tumors [89]. Besides that, it was reported that they may also experience greater toxicity from anticancer drugs. Patients with obesity who received the proper chemotherapy dose presented increased treatment-related toxicity, mainly high-grade hematological toxicities, and did not present any difference in overall survival (OS) [90]. More than that, it has been shown that adipocytes are capable of accumulating lipophilic chemotherapeutic agents, altering their distribution, and increasing enzymes that metabolize chemotherapeutic drugs. A profile that is intensified during obesity-related reduced pharmacological effectiveness, which in conjunction with greater susceptibility to tumor resistance, is associated with a worse outcome [91]. All these factors become preponderant for the therapeutic process. The increase in toxicity in the absence of positive results becomes an aggravating factor for the early discontinuation of the therapy.

Several studies have pointed to antagonistic roles that can be played by obesity across different neoplastic stages [92]. Obesity is a risk factor for tumor development, having a fundamental role in the maintenance of the inflammatory condition and also being reported as an essential factor for the onset of events such as metastasis and neoplastic recurrence [93]. However, in a contradictory and surprising way, patients impacted by obesity tend to present better prognosis for specific tumor types when compared with lean individuals [92]. Obesity-affected patients with lung cancer, renal cell carcinoma, nasopharyngeal carcinoma, and melanoma had better prognosis, including improved OS [79].

In contrast, cachexia-associated tumors tend to cope with worse disease outcome. Cancer-associated cachexia (CAC) is an irreversible metabolic syndrome characterized by loss of skeletal muscle (sarcopenia) that may or may not be associated with fat loss, and cannot be fully reversed nutritionally [94]. This syndrome is common in aggressive tumors in advanced stages and is associated with a worse prognosis, progressive functional impairment, high rates of complications, chemotherapy resistance, and high mortality in neoplastic patients [95].

An enhanced inflammatory profile is one of the main features of cachexia and it is well established that increased circulating levels of cytokines, such as TNF-α, IL-1β, IL-6, and IFNγ, secreted by both immune and non-immune cells, including tumor cells, modulate pathways related with several catabolic processes in skeletal muscle and adipose tissue [96]. Although the role of immune cells in cachexia is complex and remains not fully understood, recent research has brought better enlightenment about the influence of specific immune cell types, such as macrophages, neutrophils, microglia, myeloid-derived suppressor cells, and T cells, in CAC progression [97].

Specifically, in AT, resident immune cells, such as macrophages, can perform paradoxical roles depending on the tumor type. Erdem and colleagues demonstrated a protective role of adipose tissue macrophages (ATM) in an intercrossed transgenic murine model that genetically induces hepatocellular carcinoma (HCC) and also presents a myeloid-specific deficiency in hypoxia-inducible factor 1α (HIF1α). It was observed that an impaired myeloid cell-mediated inflammation, due to the HIF-1α knockout, promoted augmented AT depletion in parallel to a decrease of ATM abundance, indicating that macrophages can play important roles in HCC-mediated AT loss in this context [98]. In contrast, Lu and others showed that ATM and tumor-associated macrophages (TAM) may contribute to the development of CAC in pancreatic ductal adenocarcinoma (PDAC). In this study, an antibody that neutralizes the proinflammatory cytokine IL-20 was used in a transgenic and orthotopic PDAC murine model. The authors observed that inhibition of IL-20 was able to reduce tumor size, attenuate CAC symptoms, and decrease tumor PD-L1 expression. In addition, treatment with anti-IL20 induced a decrease of F4/80+IL-20+ macrophage infiltration in the epididymal AT in the transgenic PDAC model, and diminished M2-like polarization of TAM *in vitro* and in *in vivo* orthotopic PDAC model. In this case, reduction of macrophage infiltration was associated with better prognosis and increased body weight and lean mass [99].

## Immunotherapy: immune checkpoint blockade (ICB)

The use of chemotherapy, as much effective as they are, induces non-specific cytotoxicity, a relevant clinical barrier to the patient’s physical and emotional well-being, and demonstrates the need for an improvement in oncological therapies [100]. Discoveries in the area of molecular biology and cancer pathogenesis enabled the development of more effective approaches and technologies for cancer therapy [101]. Biological therapies arise from the deeper characterization of the molecular and physiological particularities of the tumor cell and its interaction with the environment in which it is inserted. The identification of tumor molecular targets (tumor-associated antigens or tumor-specific antigens), tumor mutations burden, neoantigens, defective DNA mismatch repair, microsatellite, and tumor-associated cells was essential for the development of molecular targeted therapies [102].

Immunotherapy is the class of molecular targeted therapy that aims to aid the host’s defense cells to identify and eliminate tumor cells as well as induce the formation of immunological memory, which reduces the susceptibility of tumor recurrence. This therapy has gained prominence in the cancer treatment landscape due to the success observed in pre-clinical and clinical trials for melanoma, non-small-cell lung cancer, Hodgkin lymphoma, gastric cancer, bladder cancer, head, and neck, among others [103]. Immunotherapy can be categorized into four classes of treatments: anti-tumor vaccines, oncolytic virus, adoptive transfer of T cells, and therapy with monoclonal antibodies, with emphasis on immune checkpoint blockade (ICB) [104].

ICB is a class of antibody therapies that explores the expression of immune checkpoints (ICs). Although ICs are receptors that promote inhibitory or stimulatory signals, ICB therapy acts exclusively on inhibitory receptors, such as Cytotoxic T-lymphocyte-associated antigen 4 (CTLA-4), Programmed death 1 (PD-1), Lymphocyte activation gene 3 protein (LAG3), T-cell immunoglobulin and mucin domain 3 (TIM-3). ICs activation does not occur spontaneously and depends on the interaction with their ligands, CD80/CD86, PD-L1, major histocompatibility complex (MHC) class II (MHCII), galectin-9, respectively [105]. These ligands can be expressed in antigen-presenting cells, mesenchymal stem cells, bone marrow-derived mast cells, and tumor cells in circumstances where prolonged activation and exacerbated activity of immune cells may pose a risk to the individual. In this scenario, these ICs are expressed and activated, inducing a negative regulation of the activity of immune cells, such as NK cells, T and B lymphocytes, favoring self-tolerance and preventing these cells from responding in a disorderly and indiscriminate way [106].

Some types of cancer can use the immune self-tolerance mechanism as an evasion tool, stimulating the expression of ICs and inhibiting the anti-tumor response of T cells. Antibodies, anti-ICs, and anti-IC ligands are administered to patients and selectively bind to the correspondent target, inhibiting their signaling activity. Therefore, the high expression of inhibitory IC and its ligand is essential for the proper performance of ICB therapies. The anergy phenotype is not necessarily associated with high IC expression. In contrast, the exhaustion phenotype is characterized by the increased expression of expressive IC inhibitors such as PD-1 and CTLA-4, which makes this phenotype the ideal target in CD8+ T cells. Thus, neoplasia that presents a high expression of ligands in tumor cells, such as PD-L1, as well as a high rate of tumor-infiltrating CD8+ T cells with high expression of inhibitory ICs are linked with a better response to treatments with ICB [107].

Currently, ICB, especially anti-PD1 and anti-PDL1 antibodies, represent about 2/3 of cancer therapy trials, being applicable and obtaining optimistic results alone or in combination with conventional therapies for at least 50 types of cancer, in addition to having awarded its developers the Nobel Prize in 2018 [108]. There are only two classes of ICB-targeting therapies approved by the FDA: anti-CTLA-4, and anti-PD-1/PD-L1 (PD-1 ligand 1). In 2011, FDA approved the first ICB therapy: Ipilimumab, an anti-CTLA-4 drug, designated for melanoma metastatic treatment [109]. From this, in 2014, ICB gained a second class of target, PD-1/PD-L1, with the approval of pembrolizumab and nivolumab, also for melanoma treatment [110]. Until this present review, there are approximately seven FDA-approved anti-PD-1/PD-L1 drugs and one anti-CTLA-4 drug [111]. These drugs cover a variety of cancer types, such as melanoma, non-small-cell lung cancer (NSCLC), renal cell carcinoma (RCC), gastric cancer, hepatocellular carcinoma, colorectal cancer, urothelial cancer, head and neck cancer, among others, being mainly indicated for refractory tumors or in advanced and metastatic stages [112]. Clinical trials with the use of ICB administered alone or in association with other therapies have relevant results associated with improvement in parameters, such as progression-free survival (PFS) and OS, even when compared to conventional therapies such as chemotherapy [113].

However, despite great outcomes obtained from ICB therapy, this treatment has presented limitations, and a significant proportion of patients do not benefit from the positive results provided by ICB therapy [114]. Limitations include immune-related adverse events (irAEs) and resistance to the therapy by patients, which can occur initially or during the treatment. ICB responsiveness is associated with tumor relapse [115]. ICB resistance may be associated with the tumor profile that presents a low number of tumor-infiltrating T cells and the downregulation of immune checkpoints both in the tumor and in the T cells, a profile often exhibited by low-immunogenic tumors [116].

## Obesity and ICB: the obesity paradox

**Figure 2.**
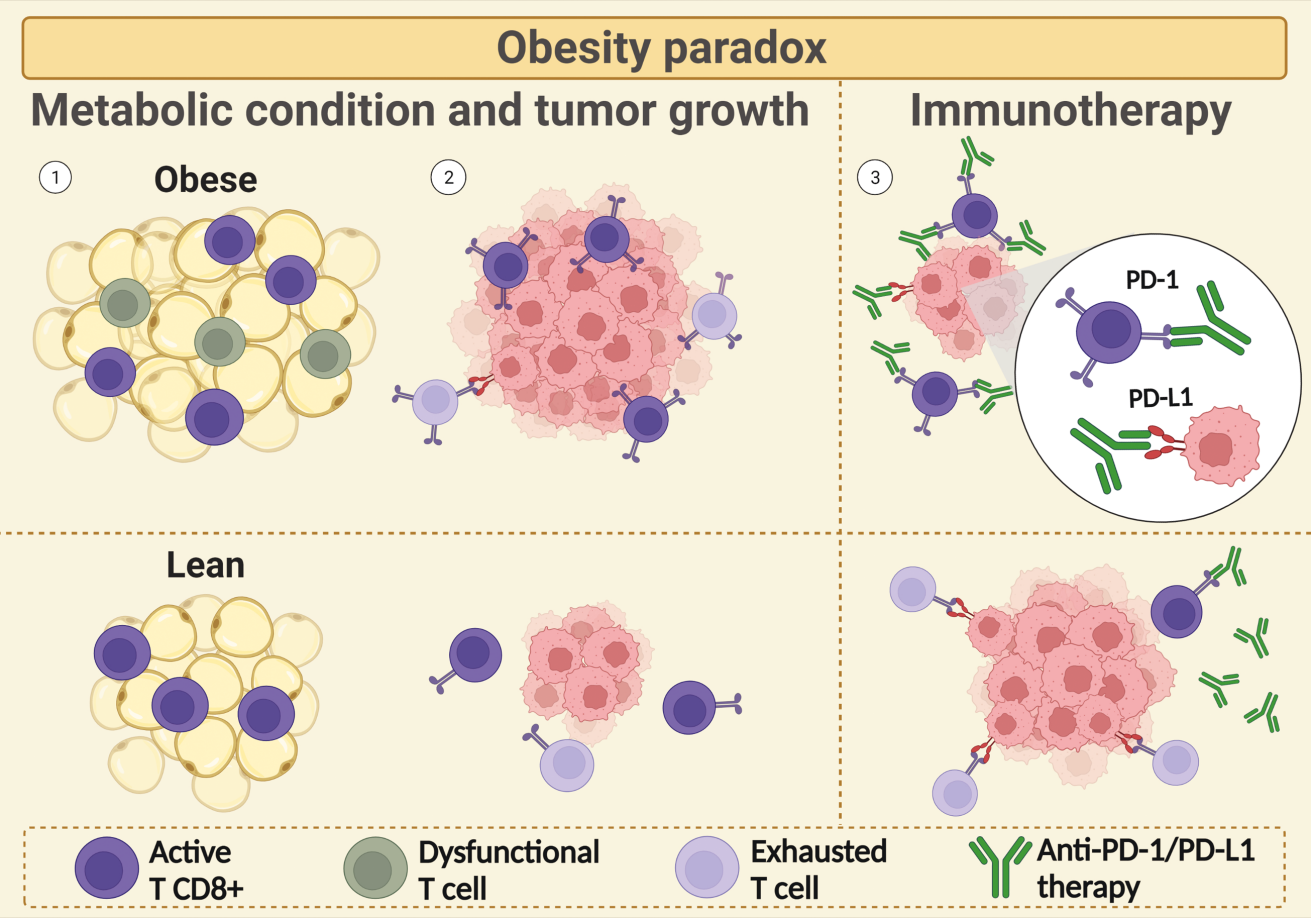
The obesity paradox. The obesity paradox represented above consists of increased tumor growth in obese patients when compared to lean, but also a better response to immunotherapy. (1) This apparently contradictory relation is explained by the abundance of dysfunctional T cells in patients with obesity. (2) This leads to an increased amount of T cells displaying an abundant expression of inhibitor receptors, such as PD-1, that, in continuous interaction with tumor cells, lead to T cell exhaustion, favoring immune evasion and tumor growth. (3) On the other hand, the augmented expression of these receptors in obesity affected-subjects allows anti-PD-1/PD-L1 therapy and other immune checkpoints blockade-based immunotherapy to diminish this T cell–tumor cell interaction, maintaining T CD8+ lymphocytes active and able to target tumor cells.

Surprisingly, BMI-classified patients with overweight or obesity tend to experience a better response to ICB than lean patients (Fig. 2) [117, 118]. Recent studies have shown that a high BMI is associated with improved PFS and OS in ICB-treated patients with metastasis in more than 20 types of cancers. Maslov and others identified that subjects presenting overweight or obesity affected by major advanced-stage cancers submitted to ICB therapy had a median PFS of 287 and 479 days, respectively, while patients in the normal weight range had a median of 128 days. The same extends to the operating system, in which patients with clinical overweight and obesity exhibited a PFS median of 462 days and 751 days, respectively, while patients with normal weight exhibited 281 days in the context of ICB treatment. In addition, secondary obesity patients display 48% less risk of progression or death from the tumor compared to patients with normal BMI [119]. An observational study showed that patients with melanoma and high BMI treated with ipilimumab (anti-CTLA-4 antibody) had a greater response and less susceptibility to metastasis to the brain, in addition to having a longer OS [120]. A meta-analysis study investigated the correlation between BMI and response to ICB treatments of approximately 4090 patients with solid tumors, especially lung cancer and melanoma, from 16 retrospective studies. Higher PFS and OS of patients with high BMI compared to patients with low BMI was observed. In addition, a reduction in the risk of death by 3.6% was observed for every 1 kg/m^2^ increase in BMI [121].

A multicohort analysis aimed to evaluate the correlation between BMI and therapeutic outcomes of targeted therapy, immunotherapy, and chemotherapy in patients with metastatic melanoma. The cohort included 538 patients treated with ipilimumab or pembrolizumab, nivolumab, or atezolizumab (anti-PD-1/PD-L1 antibodies) and, corroborating with other studies, suggested a better response associated with increased PFS and OS of patients with high BMI compared to lean patients. On the other hand, the same association between high BMI and improved response to treatment was not observed for other treatments [122]. In a clinical trial that compared the impact of BMI on the prognosis of patients treated with ICB or chemotherapy, 1,434 male patients with NSCLC were treated with atezolizumab (anti-PD-1/PD-L1) or docetaxel, a chemotherapy agent. Obesity was observed to substantially improve PFS and OS in patients treated with atezolizumab, an outcome associated with increased PD-L1 expression in these patients’ tumors. But the same was not observed for patients who received docetaxel. In this study, the side effects observed were not related to BMI [83].

The obese phenotype copes with T lymphocyte dysfunction, a state characterized by a hyporesponsive profile, associated with loss of effector, proliferative capacity, and reduced production of cytokines, TNF-α and IFN-γ, in conditions that can be classified as anergy or exhaustion [123]. Anergy is a profile characterized by an inadequate activation of CD8+ T cells due to low co-stimulatory signaling, mediated by stimulatory IC, such as CD-28 and OX-40, associated or not with an increase of inhibitory signal mediated by inhibitory IC expression. Anergy phenotype is frequently observed in the early stages of the tumor progression [124]. In contrast, exhaustion is a profile exhibited by CD8+ T cells after chronic and persistent exposure to antigens, which in a self-tolerance mechanism, triggers a remarkable increase of inhibitory IC expression, including PD-1 and CTLA-4, to suppress their activity. Exhausted CD8+ T cells are frequently seen in more advanced tumors and can be divided into two populations: progenitor exhausted or terminally exhausted [125]. Miller and colleagues suggest that the efficacy of therapies with PD-1 blockade is associated with different subpopulations and functionalities of exhausted CD8+ T cells, since progenitor exhausted CD8+ T lymphocytes can recover their effector activity, unlike terminally exhausted ones [126].

The excess adiposity may promote a greater expression of PD-1 in effector CD8+ T lymphocytes, promoting a better response to ICB therapies. Zhang and others informed that obesity was related to a decrease in the effector activity of CD8+ T cells in PyMT mice, which is a rodent model that spontaneously develops breast tumors, through a metabolic change consisting of increased oxidative phosphorylation (FA oxidation) and reduced glycolysis [127]. Moreover, Kado and colleagues demonstrated that the high-fat diet was able to induce a shift in the CD8+ T cell profile to an exhausted CD8+ PD-1+ T cell profile in animals with breast cancer. In the same line, obese mice impacted by melanoma have been observed to have better responses to ICB therapies. These findings extended to clinical data associated with other tumors, such as NSCLC, RCC, ovarian cancer, and others [118]. The impact of obesity on PD-1 T lymphocyte levels may explain the increased effectiveness of ICB therapy against certain tumors displayed by this population.

A paradoxical predictor for anti-tumoral response and response to ICB therapy is a high rate of glycolytic activity exhibited by tumor cells. The Warburg effect is an adaptive mechanism acquired by tumor cells that gives them a selective advantage over other cells in the environment. Healthy cells use glucose as the substrate to obtain energy, adenosine triphosphate (ATP), through the Citric Acid Cycle (TCA) pathways and oxidative phosphorylation (OXPHOS). However, in the 1920s, Otto Warburg observed an excessive consumption of glucose by tumor cells compared to healthy cells. Moreover, he observed that consumed glucose was converted to lactate even in the presence of oxygen in a phenomenon that became known as the Warburg effect or aerobic glycolysis [128]. Several hypotheses have been developed since that to explain the metabolic shift to another pathway. Recent studies have demonstrated that the Warburg effect appears to provide advantages that support the growth, proliferation, and evasion of tumor cells [129].

The Warburg effect has direct and indirect roles on the immune system that favor tumor progression. Colegio and colleagues demonstrated that tumor-derived lactate acts to polarize tumor-associated macrophages in an M2 profile by impacting the expression of HIF1-α [130]. In addition, the high consumption of glucose by tumor cells diminishes their bioavailability in the environment, which has a direct impact on activation and effector activity, such as the production of IFN-γ from tumor-infiltrating lymphocytes (TIL) [131]. High glycolytic activity can be considered a good predictor of the response to anti-PD-1/ PD-L1 immunotherapy since the increase in aerobic glycolysis is associated with the increased expression of PD-L1 in the tumor [132].

In addition, estrogen circulating amounts activity can also be placed as a possible mechanism for the obesity paradox, especially for melanoma patients. The female gender has long been associated with favorable melanoma outcomes when compared to males. Although melanoma is recognized as non-hormone cancer, several studies have indicated that sex hormone signaling, more specifically estrogen, is responsible for better prognosis and improved therapies responses of this tumor [133]. Estrogen is a sex steroid hormone more abundant in females. This hormone directly promotes melanocyte differentiation, increases the production of melanin, and decreases proliferative capacity through a non-classical Gs-coupled G protein-coupled receptor (GPCR) called G-protein coupled estrogen receptor (GPER). Moreover, GPER activation depletes c-Myc protein, a transcription factor that regulates several genes involved in essential cellular pathways, including cell growth, proliferation, survival, differentiation, and immune checkpoint expression, such as PD-L1, on the tumor [134]. Natale and colleagues demonstrated that GPER activation of B16-F10 melanoma cells inhibits growth and favors higher susceptibility of these cells to immunotherapy anti-PD-1 antibody *in vitro*. A similar phenomenon was observed in melanoma-bearing mice that received GPER agonists associated with anti-PD-1 antibody. G-1 pretreatment of human melanoma cells inhibited tumor growth in mice and increased the innate and adaptative anti-tumor immune cells infiltration, such as NK and T CD8+ cells within the tumor, demonstrating a great potential of GPER agonist as a cancer immunotherapy adjuvant [135].

In the same way, it is hypothesized that these same biological phenomena arising from estrogens are responsible for the successful response of immunotherapy in obese male patients with melanoma. Obese men present higher expression of aromatase, an enzyme that irreversibly converts androgens, such as testosterone, into estrogen. Aromatase expression is proportional to body fat mass and promotes a significant increase of circulating estrogen in obese individuals [136]. However, it is still not possible to state that the activation of GPER, which has been shown to play a central role in the prognosis of female melanoma patients, is responsible for the efficacy of ICB observed in obese men. To this end, further investigation is required to obtain more concrete explanations.

Nevertheless, recent studies have brought a new perspective to the obesity paradox in ICB antibody treatments. Similarly, it has been shown that overweight or obese phenotypes are also linked with an increased likelihood of experiencing different degrees of irAEs [137]. Cortellini and others investigated 1070 patients with NSCLC, melanoma, RCC, or other advanced-stage cancers who received anti-PD-1/PD-L1 treatments and observed a correlation between high BMI and the development of irAEs that included cutaneous, endocrine, gastrointestinal, liver, among others, adverse events. Particularly, obese patients experienced a high incidence of rheumatic and pulmonary irAEs compared to patients with normal BMI. Such complications are often followed by discontinuation of treatment and therefore further investigations are needed to characterize the benefits and risks associated with BMI and treatment with ICB [138]. Taken all together, the need for better characterizing other parameters that may influence the different results regarding efficacy versus adverse events obtained in high BMI neoplastic patients treated with ICB is evident.

Body composition is a large spectrum that presents several intermediate phenotypes between these extremes. Among them, there is a phenotype called sarcopenic obesity (SO). SO is characterized by a significant loss of lean mass camouflaged by little or no change in adiposity. This phenotype poses a great challenge regarding cancer therapy [139]. Studies revealed that about 20% of obese patients with advanced or metastatic cancer presented SO [140]. Nonetheless, little is known about the impact of SO in immunotherapy. Until the present review, no clinical trials have been published about the relation between SO and immunotherapy efficacy or toxicity. However, Heidelberger and colleagues performed a preclinical study with 77 patients with advanced melanoma, including 13 (19%) sarcopenic overweight patients treated with the anti-PD1 checkpoint inhibitors nivolumab or pembrolizumab in doses of 3 mg/kg every 2 weeks and 2 mg/kg-dose every 3 weeks, respectively. It was observed that sarcopenic female patients presented 6.5-fold more anti-PD1-related early acute limiting toxicity and did not present any improvement in the anti-tumor response. The authors hypothesized that it could be related to weight-based dosing, which indicates a high drug dose administration, once it is assumed that pharmacokinetic parameters are altered in patients with high BMI and drug distribution may be impaired due to loss of lean mass in sarcopenic patients [141]

## Conclusion

It is undeniable that ATs play fundamental roles in the individual’s immune and inflammatory modulations. The recognition of ATs as immune organs, in addition to continuous discoveries that reinforce the intrinsic relationship of this issue with the immune system, has brought new perspectives to the investigation of AT’s role in several metabolic, infectious and neoplastic diseases. ATs’ composition and their plastic phenotype give these tissues the ability to substantially modulate aspects such as onset progression and prognosis of various pathologies. Therefore, the increase in observational studies using the ATs’ profile as a scalable factor in favor of promoting the improvement of local and systemic conditions is not surprising.

As we showed in the present review, WAT and BAT functions and their impact on system physiology are intimately dependent on their immune cell components. The proinflammatory characteristics of ATs derived from individuals affected by obesity contribute to metabolic and immune systemic disturbances that associate this group with a scenario of a greater propensity for the development of, in general, a worse prognosis of fatal diseases, including cancer. Recent data have clearly shown the increase in obesity-related tumors, contrary to what has been observed about the incidence of tumors in general, which has shown a downward trend in the world. Tumor progression and mortality rate are also positively associated with high BMI. However, with the use of technologies and innovations in the development of new cancer treatments, and an antagonistic phenomenon called ‘The obesity paradox’ was observed. In a specific strand of immunotherapy, antibodies blocking immune checkpoints, obesity appeared to be related to better prognosis with increased PFS and OS. The main hypothesis associated with this phenomenon is the ability of adipose tissue to modulate the phenotype of immune cells, especially CD8+ PD-1+ T cells, in a dysfunctional profile, but responsive to treatment due to a higher expression of the PD-1 target molecule.

The phenomenon is intriguing but not homogeneous. Some clinical studies have shown that in parallel to the greater efficacy of ICB therapy in overweight and obese patients, higher incidences of adverse events related to the therapy have also been observed. All these findings highlight the need for further investigation and characterization of adjacent processes that may be influencing this phenomenon observed in neoplastic patients with high BMI.

## Data Availability

Not applicable.

## Abbreviations

AT: Adipose tissue
ATM: Adipose tissue macrophages
ATP: Adenosine triphosphate
BMI: Body mass index
Breg: B regulatory
BAT: Brown adipose tissue
CAC: Cancer-associated cachexia
cAMP: Cyclic adenosine monophosphate
CCL: CC chemokine ligands
CD: Cluster of differentiation
CDC: Centers for Disease Control and Prevention
CLS: Crown-like structure
CTLA-4: Cytotoxic T-lymphocyte-associated antigen 4
DNA: Deoxyribonucleic acid
ER: Endoplasmic reticulum
ET-1: Endothelin-1
FAs: Fatty acids
FDA: Food & Drug Administration
FFA: Free fatty acids
FGF21: Fibroblast growth factor 21
GLUT1: Glucose receptor 1
GPCR: Gs-coupled G protein-coupled receptor
GPER: G-protein coupled estrogen receptor
HCC: Hepatocellular carcinoma
HIF1α: Hypoxia-inducible factor 1α
IARC: International Agency of Research on Cancer
IC: Immune checkpoint
ICB: Immune checkpoint blockade
IFN: Interferon
Ig: Immunoglobulin
IL: Interleukin
ILCs: Innate lymphoid cells
iNKT: Invariant natural killer T
IR: Insulin resistance
irAEs: Immune-related adverse events
LAG3: Lymphocyte activation gene 3 protein
MHC: Major histocompatibility complex
M1: Classically activated macrophages
M2: Alternatively activated macrophages
NSCLC: Non-small-cell lung cancer
OS: Overall survival
OXPHOS: Oxidative phosphorylation
PDAC: Pancreatic ductal adenocarcinoma
PD-L1: Programmed death-ligand 1
PD-1: Programmed cell death-1
PFS: Progression-free survival
RCC: Renal cell carcinoma
ROS: Reactive oxygen species
scWAT: Subcutaneous WAT
SLIT2-C: SLIT2 protein
SO: Sarcopenic obesity
TAM: Tumor-associated macrophages
TCA: Tricarboxylic acid cycle
TCR: T-cell receptor
Teff: Effector T
TGF-β: Transforming growth factor-beta
Th: T helper
TIL: Tumor-infiltrating lymphocytes
TIM-3: T-cell immunoglobulin and mucin domain 3
TNF: Tumor necrosis factor
Treg: T regulatory
TGs: Triglycerides
T2D: type 2 diabetes
UCP1: Uncoupling protein 1
vWAT: Visceral white adipose tissue
WAT: White adipose tissue
WHO: World Health Organization
β2AR: Norepinephrine-beta2-adrenergic receptor.
